# Editorial: Preterm brain injury: Understanding injurious processes and new strategies for promoting neuroprotection and neuro-repair

**DOI:** 10.3389/fphys.2022.994521

**Published:** 2022-08-29

**Authors:** Megan Finch-Edmondson, Rod W. Hunt, Jens Bo Nielsen, Madison C. B. Paton

**Affiliations:** ^1^ Cerebral Palsy Alliance Research Institute, Speciality of Child and Adolescent Health, Sydney Medical School, Faculty of Medicine and Health, The University of Sydney, Sydney, NSW, Australia; ^2^ Monash Newborn Research, Department of Paediatrics, Monash University, Melbourne, VIC, Australia; ^3^ Department of Neuroscience, University of Copenhagen, Copenhagen, Denmark

**Keywords:** preterm brain injury, neuroinflammation, neuroprotection, neuroplasticity, novel interventions

Brain development is highly complex, requiring precise regulation to ensure correct organisation and functioning. Preclinical and clinical research have been integral in advancing our knowledge of brain development, as well as the consequences when normal development is disrupted, such as occurs following preterm birth. Every year, approximately 15 million babies are born preterm worldwide, and rates of prematurity are increasing in many countries (March of Dimes, PMNCH, Save the Children, WHO, 2012). Preterm birth is a significant cause of morbidity and mortality, with survivors often facing a lifetime of disability, including motor impairments, learning and behavioural difficulties, and visual and hearing problems (March of Dimes, PMNCH, Save the Children, WHO, 2012).

A number of factors, both antenatal and postnatal, have been identified that can contribute to preterm brain injury. These include infection, hypoxia-ischemia, mechanical ventilation and hemodynamic instability. Whilst mechanisms underlying injury to the preterm brain are complex, preclinical and clinical research have helped identify a number of key injurious processes of interest. These include inflammation, oxidative stress, cell death and impaired cell proliferation and differentiation (Back, 2017).

There are currently limited treatment options available to improve outcomes following preterm brain injury, and none that specifically target neuroprotection or neuro-repair. Despite this, ongoing research using various preclinical models of brain injury, as well as clinical studies of preterm cohorts, continue to improve our understanding of the injurious processes associated with preterm birth. Moreover, new strategies and interventions that might protect the vulnerable preterm brain, or even promote brain repair following injury, are being discovered. These include pharmacological and biological therapies as well as neuroplasticity-inducing interventions such as rehabilitation, environmental enrichment, and other innovative approaches (Finch-Edmondson et al., 2019). Moreover, new strategies that optimise perinatal care and newborn transition, as well as early management to reduce deleterious symptoms of preterm birth continue to be revealed.

This Research Topic aimed to showcase new findings or highlight novel ideas in the field of preterm brain injury, neuroinflammation and innovative approaches to protect and/or repair the injured preterm brain. This issue comprises six papers, including both original studies and review articles, that provide a valuable contribution to research in preterm brain injury.

Preterm newborns commonly have a compromised ability to breathe after birth and may require respiratory support. Moreover, a substantial proportion of preterm babies are exposed to *in utero* inflammation. Using a fetal preterm sheep model, Stojanovska et al. investigated the association between antenatal inflammation, the inflammatory mediator prostaglandin E2, and inhibition of fetal breathing. They demonstrate that LPS-mediated inflammation led to increased levels of prostaglandin E2, inhibited fetal breathing movements and was associated with neuropathological changes within the brainstem respiratory centres. Importantly, their results identify prostaglandin E2 as a potential therapeutic target for improving respiratory drive in preterm born infants. Successful targeting of prostaglandin E2 may reduce the need for potentially injurious respiratory support and thus contribute to improving neurodevelopmental outcomes.

Complementing the study by Stojanovska et al., in their original research article, Chan et al. explore the relative roles of the inflammatory and haemodynamic pathways on ventilation induced brain injury in the preterm lamb. Notably, the authors discovered that the haemodynamic response, namely the irregular variations in blood flow following the removal of the placental circulation, can produce an additional inflammatory response, exacerbating ventilation-induced brain injury which may contribute to adverse outcomes. Procedures such as physiological-based cord clamping are suggested as a strategy to reduce injurious physiological responses to ventilation in preterm infants.

Seizures are the most common neonatal neurological event following preterm birth and perinatal brain injury (Spagnoli et al., 2018). Ganaxolone is a GABA_A_ receptor modulator which was recently approved for the treatment of seizures associated with cyclin-dependent kinase-like 5 (CDKL5) deficiency disorder (Lamb, 2022). Ganaxolone also shows promise as a novel therapeutic to reduce preterm brain injury (Shaw et al., 2019). In a follow-up to their pilot study, Shaw et al. focused on dose-optimisation for safety with maximal therapeutic gain. Using their established preterm guinea pig model, the authors tested three doses of ganaxolone for effects on physical health and well-being, as well as hippocampal neurodevelopment. Promisingly, this study identified ganaxolone doses with a favourable tolerability and safety profile, paving the way for future longer-term studies examining functional outcomes.

Although animal models are a very important and useful tool for understanding the pathophysiology of human disease, the challenges associated with attempting to faithfully recapitulate human conditions are well known. In their review, Favrais et al. focus specifically on the complexity of oligodendrocyte maturation, proliferation and differentiation following inflammatory stimuli in two rodent models of preterm brain injury. Interestingly, the authors found differential effects on oligodendrocytes in response to two commonly used stimuli (*E. coli* liposaccharide and Interleukin-1β). This finding serves as a timely reminder to researchers who may be utilizing animal models to support the discovery of new neuroprotective strategies for preterm brain injury.

Finally, in their review, Gunn-Charlton shines a light on the clinical complexities pertaining to the frequent co-existence of prematurity and congenital anomalies requiring surgery during the neonatal period, and discusses their combined influence on the risk of brain injury and neurodevelopmental impairment. Exploring these two significant risk factors for brain injury in detail, Gunn-Charlton calls for greater collaboration between the disciplines of perinatal/preterm care, cardiac intensive care and surgical neonatal care, with the common goal of improving outcomes for these high-risk infants.

Together, these six articles represent an important addition to the scientific literature in uncovering factors that contribute to the pathogenesis of preterm brain injury and new strategies to better current perinatal care. Future research is required to progress interventions and perinatal care with an aim to prevent preterm brain injury and continue to improve neurodevelopmental outcomes.
